# Post-Invasion Recovery of Plant Communities Colonised by *Gunnera tinctoria* after Mechanical Removal or Herbicide Application and Its Interaction with an Extreme Weather Event

**DOI:** 10.3390/plants11091224

**Published:** 2022-04-30

**Authors:** Maurício Cruz Mantoani, Bruce Arthur Osborne

**Affiliations:** 1 UCD School of Biology and Environmental Science, and UCD Earth Institute, University College Dublin (UCD), Belfield, Dublin 4, D04 V1W8 Dublin, Ireland; 2 Institute of Astronomy, Geophysics and Atmospheric Science (IAG), University of São Paulo (USP), São Paulo 05508-090, Brazil

**Keywords:** climatic extremes, competition, ecological restoration, ecosystem resilience, environmental thresholds, glyphosate, invasive alien plants, *Juncus effusus*

## Abstract

The interventions that are required for both the control and post-invasion restoration of native plant communities depends on several factors, including the efficacy of the measures that are used and how these interact with environmental factors. Here, we report on the results of an experiment on the effects of mechanical removal and herbicide application on the invasive plant *Gunnera tinctoria* and how an extreme weather event impacted on the invader and on the recovery of native coastal grassland communities. Both removal protocols were largely effective in eradicating mature plants, but the mechanical removal treatment resulted in a major increase in the number of *G. tinctoria* seedlings, which was exacerbated by the extreme event. Nine months after removal, the number of native species had recovered to *c.* 80% of that in uninvaded grasslands. In contrast to seedlings, mature plants of *G. tinctoria* showed a significant reduction in above-ground production after the extreme weather event, although these had largely recovered after six months. Overall, our results indicate that post-control restoration of the plant community may be possible without further significant management interventions. Nevertheless, since some invasive plants survived, further monitoring is required to ensure that recolonisation does not occur.

## 1. Introduction

The effective long-term management of ecosystems colonised by alien plant species includes not only the successful removal of the invader but also the restoration of the previously invaded community [1]. Ideally, the removal of the plant invader would lead to the natural regeneration of the native plant community, although this could depend on several factors that have been long studied in restoration ecology [2,3], including the influence of the prevailing climatic conditions and the reservoir of viable seeds remaining in the seed bank. However, it is increasingly recognised that future changes in climate may also interfere with restoration initiatives [4]. Although these could impact on the efficacy or on the timing of management measures that are currently used to control invasive species [4,5,6], this has received little attention. 

Given that the numbers of invasive alien species are still increasing [7], this suggests that management programmes have been largely ineffective on a global scale despite individual examples of successful regional scale interventions [1]. There are a variety of possible reasons why many management interventions may have not been successful. These might include the use of inadequate or short-term control measures, a failure to account for post-invasion recruitment and/or establishment from the soil seed bank. Climatic factors, including extreme weather events, may also impact on the efficacy of the approaches used [4]. 

Overall, climate change may often benefit introduced species, and the increased occurrence of extreme weather events may be particularly important [8]. Evidence shows that these may favour plant invaders by reducing the resilience and/or the recovery of ecosystems [9,10,11,12]. For instance, it was found that there was a higher mortality rate of native trees in invaded areas due to the synergistic impact of a heatwave and the presence of an invasive shrub, *Cistus ladanifer* [13]. There is also evidence that extreme weather events can alter habitat structure [14], interacting with anthropogenic disturbances [14,15] and interfering with restoration initiatives [16,17,18]. Such climatic extremes can have a wide range of effects on ecosystems and ecosystem processes that could, in turn, impact on management and restoration measures. These include: (*i*) modifications in species phenology [10,19,20,21,22]; (*ii*) alterations in physiological processes, such as carbon assimilation and biomass production [21,23]; (*iii*) shifts in plant community composition [8,9,10]; (*iv*) modification in fire regimes or fire severity [9,24,25]; and (*v*) alterations in nutrient cycling and carbon sequestration [10,26].

The introduced species *Gunnera tinctoria* is an important N-fixing invasive plant species that is associated with significant detrimental impacts on ecosystems and communities worldwide [27] and is documented on the EU List of Invasive Alien Species of Union Concern [28]. For established invasions by *G. tinctoria*, there is a significant reduction in both the abundance and biodiversity of species present in the seed bank [29]. This could limit the successful restoration of the original community in the absence of any additional management interventions. In addition, disturbance events associated with the physical removal of large herbaceous invaders, such as *G. tinctoria*, might also create conditions that are unsuitable for the germination of native species whilst promoting colonisation by other alien or unwanted weed species (i.e., invasion meltdown) [30]. Although herbicides have been proved to be effective in controlling a wide range of alien plants, including *G. tinctoria* [31], these may result in significant legacy effects that limit the growth and germination of native species, as well as having other unwanted environmental impacts [32].

To examine the effectiveness of two commonly used control measures, we examined the impact of mechanical removal and herbicide applications on invasive populations of *G. tinctoria*, together with assessments of the post-invasion recovery of the vegetation, over two years. To verify how post-removal recovery of the vegetation was impacted by extreme weather conditions we took advantage of a country-wide event (Storm Emma), which occurred in late February to early March 2018 and resulted in temperatures plunging to below 0 °C with snowfall over a major part of Ireland, during the early part of the growing season after the initiation of plant growth. Our hypotheses were that: (*i*) plant removal would result in a poor and slow recovery of the native vegetation since *G. tinctoria* invasions are known to deplete the abundance and richness of viable seeds in the soil seed bank [27,29]; (*ii*) the use of herbicide would be more effective, in terms of controlling the plant invader, in comparison to physical removal as it would reduce any disturbance-related increases in *G. tinctoria* seedlings emerging from the soil seed bank; (*iii*) since this invasive plant is sensitive to low temperatures [33], we expected that the survival of both mature plants and seedlings would be poor after an extreme weather event, which would reduce its abundance and coverage; and, as a consequence, (*iv*) we anticipated that Storm Emma would favour native species, such as *Juncus effusus*, which presumably has a greater tolerance of low temperatures [34], aiding in the post-invasion recovery of the community.

## 2. Results

### 2.1. Species Richness

**Figure 1:**
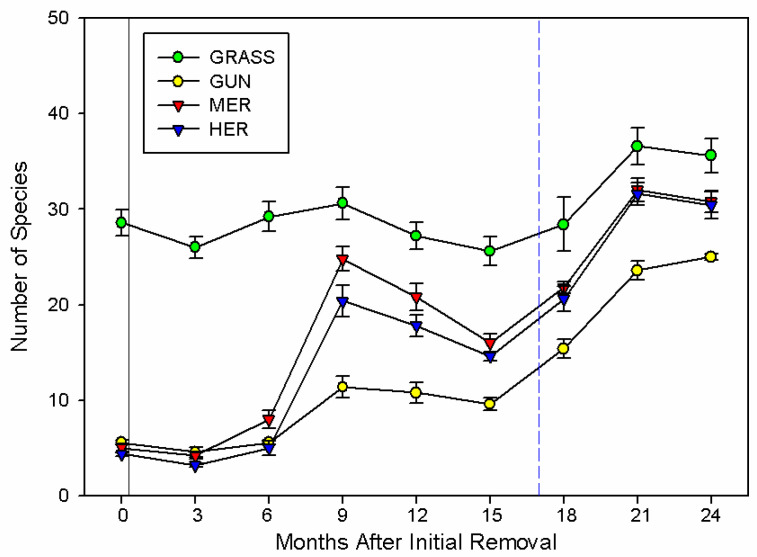
Time course of changes in the number of species per plot after the physical removal of mature *G. tinctoria* plants or the application of herbicide on Achill Island, Co., Mayo, Ireland (n = 5; mean ± SE). Legend: GRASS, uninvaded semi-natural grasslands; GUN, areas invaded by *G. tinctoria*; MER, mechanical removal; and HER, herbicide (glyphosate) application. Note: the black solid line represents the initial removal, performed in invaded areas on 29th of September 2016, and the blue dashed line represents the occurrence of the extreme weather event called Storm Emma, at the end of February and beginning of March 2018.

Species richness varied with sampling time and between treatments (F_(24,128)_ = 21.75; *p* < 0.001; Figure 1). Prior to the initial removal or herbicide application (Time 0), the number of species was fivefold lower (6 ± 0.24; 95% CI = −27.65, 18.35) in the invaded plots compared to the uninvaded grasslands (29 ± 1.36). One year after removal, there was a significant increase in species in the mechanical removal (21 ± 1.39; 95% CI = 5.35, 14.65) and herbicide-treated plots (18 ± 1.11; 95% CI = 2.35, 11.65), in comparison to invasive stands (11 ± 1.07; Figure 1). However, in comparison to the uninvaded plots, both mechanical removed (−6.4, 95% CI = −11.05, −1.75) and herbicide-treated plots (−9.4, 95% CI = −14.05, −4.75) still had lower values (Figure 1). 

After the extreme weather event, there was a major increase in the number of species in invaded plots (25 ± 0.32), with these areas having four times more species at the end of the study (95% CI = 15.42, 23.38) compared to the beginning (Figure 1). As the number of species had also increased in uninvaded grasslands (36 ± 1.78; 95% CI = 3.02, 10.98), areas where plants were physically removed (31 ± 1.16; 95% CI = 15.42, 23.38) and applied with herbicide (30 ± 1.44; 95% CI = 22.02, 29.98), *G. tinctoria* plots still had a lower species richness in comparison to the uninvaded areas (95% CI = −15.25, −5.95), physical removal (95% CI = −10.45, −1.15) or herbicide-treated plots (95% CI = −10.05, −0.75; Figure 1). 

### 2.2. Survival and Abundance of Gunnera tinctoria

**Figure 2:**
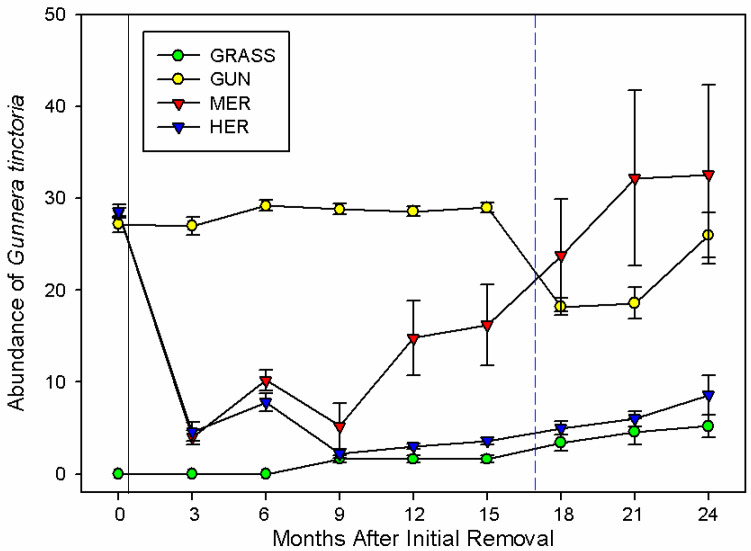
Time course of the number of mature *G. tinctoria* plants and their seedlings per plot after the physical removal of mature plants or the application of herbicide (n = 5; mean ± SE), on Achill Island, Co., Mayo, Ireland. Legend: GRASS, uninvaded semi-natural grasslands; GUN, areas invaded by *G. tinctoria*; MER, mechanical removal; and HER, herbicide (glyphosate) application. Note: the black solid line represents the initial removal, performed in invaded areas on 29th of September 2016, and the blue dashed line represents the extreme weather event called Storm Emma, at the end of February and beginning of March 2018.

The number of individual *G. tinctoria* seedlings or the ability of established rhizomes to produce above-ground shoots after the extreme weather event varied through time (F_(24,128)_ = 22.44; *p* < 0.001; Figure 2). In uninvaded semi-natural grasslands, the abundance of the plant invader went from none to an average of 5 individuals (95% CI = −4.38, 14.78) with some evidence of an increase after the extreme weather event (Figure 2). Herbicide applications significantly reduced the number of plants that survived prior to the extreme weather event, even though some plants recovered. At the end of the experiment, and after the extreme weather event, there were still significantly fewer *G. tinctoria* plants in herbicide-treated areas (9 ± 2.16; 95% CI = −29.58, −10.42) than at the beginning of it (29 ± 0.68). In contrast the number of plants in mechanically removed plots was higher than the number of plants before removal, although this was not significant (33 ± 9.78 versus 28 ± 0.51; Figure 2). 

The assessment of the effect of the extreme weather event on established plants was based on the visual appearance of the apical buds and shoots, many of which turned brown after the extreme weather event resulting in a major reduction in above-ground growth, although many plants subsequently recovered (see Material and Methods). After the extreme weather event the number of *G. tinctoria* plants in invasive stands able to produce new shoots initially declined (−9; 95% CI = −18.58, 0.58). Nonetheless, at the end of the experiment this was not significantly different (26 ± 2.47) from that prior to the extreme event (27 ± 0.86; Figure 2). If we assume that the inability of rhizomes to produce new shoots at the end of the experimental period indicates they are dead, the extreme weather event was responsible for killing an average of 11 ± 0.66 rhizomes or 37% of mature *G. tinctoria* individuals, diminishing its density from 1.16 ± 0.02 in January (15 months after the initial removal) to 0.73 ± 0.04 rhizomes m^−2^ in April 2018 (18 months after the initial removal). By the end of the experimental period, however, the resprouting of shoots from newly developed apices on the rhizome resulted in a density that was similar to that of prior to the extreme weather event (1.04 ± 0.10). 

### 2.3. Abundance and Cover of Juncus effusus

**Figure 3:**
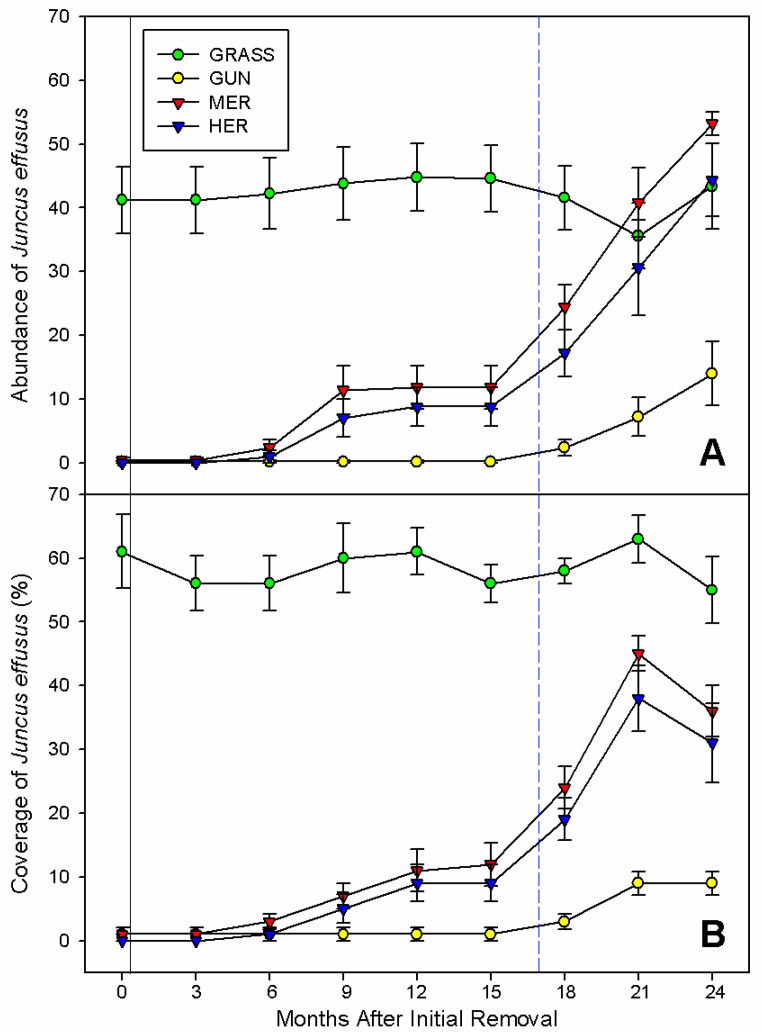
Changes in the (**A**) abundance and (**B**) coverage of *J. effusus* per plot after the physical removal or application of herbicide to mature *G. tinctoria* plants (n = 5; mean ± SE), on Achill Island, Co., Mayo, Ireland. Legend: GRASS, uninvaded semi-natural grasslands; GUN, areas invaded by *G. tinctoria*; MER, mechanical removal; and HER, herbicide (glyphosate) application. Note: the black solid line represents the initial removal, performed in invaded areas on 29th of September 2016, and the blue dashed line represents the extreme weather event called Storm Emma, at the end of February and beginning of March 2018.

The abundance of *J. effusus* varied significantly over the course of the experiment (F_(24,128)_ = 10.14; *p* < 0.001; Figure 3A). Little change in the number of plants were found in invasive stands up to the occurrence of the extreme weather event (0.20 ± 0.20). After Storm Emma, however, the number of *J. effusus* plants increased (14 ± 5.06; 95% CI = 21.02, 52.75) sharply (Figure 3A). In invasive stands, however, the number of individuals of *J. effusus* was still smaller than in the uninvaded areas (95% CI = −35.15, −24.60), mechanical removal (95% CI = −39.08, −14.57) and herbicide-treated plots (95% CI = −35.72, −13.32) plots (Figure 3A).

After physical removal or herbicide-spraying, there was initially a small increase in the number of individuals of *J. effusus*, with some evidence of a plateaux after nine months (Figure 3A). This was followed by a steep increase after the extreme weather event, with the number of plants in the physical removal and herbicide treatments increasing from almost zero to 53 ± 1.77 and 44 ± 5.71, respectively (Figure 3A). In the uninvaded grasslands, the abundance of *J. effusus* showed little variation with time (*c.* 42 plants per plot) up until the extreme weather event, where there was evidence of a decline (36 individuals) followed by a recovery, with no differences amongst sampling periods (Figure 3A). In general, variations in cover paralleled the patter described for its abundance, although there was no evidence of a decline after the extreme weather event in uninvaded grasslands, areas where invasive plants were physically removed or applied with herbicide (Figure 3B). 

### 2.4. Plant Community Similarity

Prior to the imposition of the experimental treatments the plots had a *c.* 60% similarity index, indicating some significant variability in the community composition of the invaded areas. However, the similarity between these plots and the uninvaded grassland was only *c.* 16% indicating marked differences in plant community composition (Table 1). Two years after the mechanical removal of *G. tinctoria* or the application of herbicide, and prior to the extreme weather event, the similarity index increased to similar values (*c.* 65%). After the extreme weather event, all treatments, including the invaded plots shared *c.* 80% of species with the uninvaded grassland areas (Table 1). At the end of the study, after Storm Emma, the smallest Jaccard index (*c.* 77%) was found in the comparison between the invaded and uninvaded areas, whilst the highest value was found between the mechanical removal and herbicide treatments (*c.* 94%; Table 1). 

At the beginning of the study, invaded areas had 10 or less species and were mainly colonised by *Agrostis capillaris*, *A. stolonifera*, *Equisetum arvense* and *E. fluviatile*, *Epilobium obscurum*, *Galium aparine*, *J. effusus*, *Ranunculus acris* and *R. repens*, and *Urtica dioica* (Table 2). The same species were also present in uninvaded areas, but grassland plots had 32 species that were absent under invasive stands. Native grasses such as *Alopecurus pratensis*, *Festuca ovina*, *Holcus* spp., *Lolium perenne*, *Poa* spp., as well as common Irish grasslands species, for example, *Angelica sylvestris*, *Apium nodiflorum*, *Cirsium palustre* and *C. vulgare*, *Lythrum salicaria*, *Mentha aquatica*, *Prunella vulgaris*, *Rubus fruticosus*, *Rumex* spp., and *Trifolium pratense* and *T. repens* were all present in uninvaded grasslands (Table 2). 

One year after removal, species such as *A. pratensis*, *A. sylvestris*, *A. nodiflorum*, *C. palustre* and *vulgare*, *L. salicaria*, *M. aquatica*, *Poa* spp. and *T. repens* colonised areas where the plant invader was either physically removed or sprayed with herbicide. In addition, previously uninvaded grassland plots were colonised by *G. tinctoria* (Table 2). Only after Storm Emma, did species such as *C. palustre*, *J. articulatus*, *L. salicaria*, *M. aquatica, Potentilla* spp., *P. vulgaris*, *Rumex* spp. and *T. repens*, and common grasses, for instance, *F. ovina* and *H. lanatus,* appear in previously *G. tinctoria* plots. Nevertheless, some species were only present in uninvaded areas, for instance, *Anthoxanthum odoratum*, *Callitriche stagnalis* and *Filipendula ulmaria*. In addition, *Anagallis tenella*, *Carex echinata*, *Daucus carota* and *Persicaria maculosa* were only recorded after the extreme event (Table 2). 

### 2.5. Effects of Plant Removal on Soil Environmental Variables

**Figure 4:**
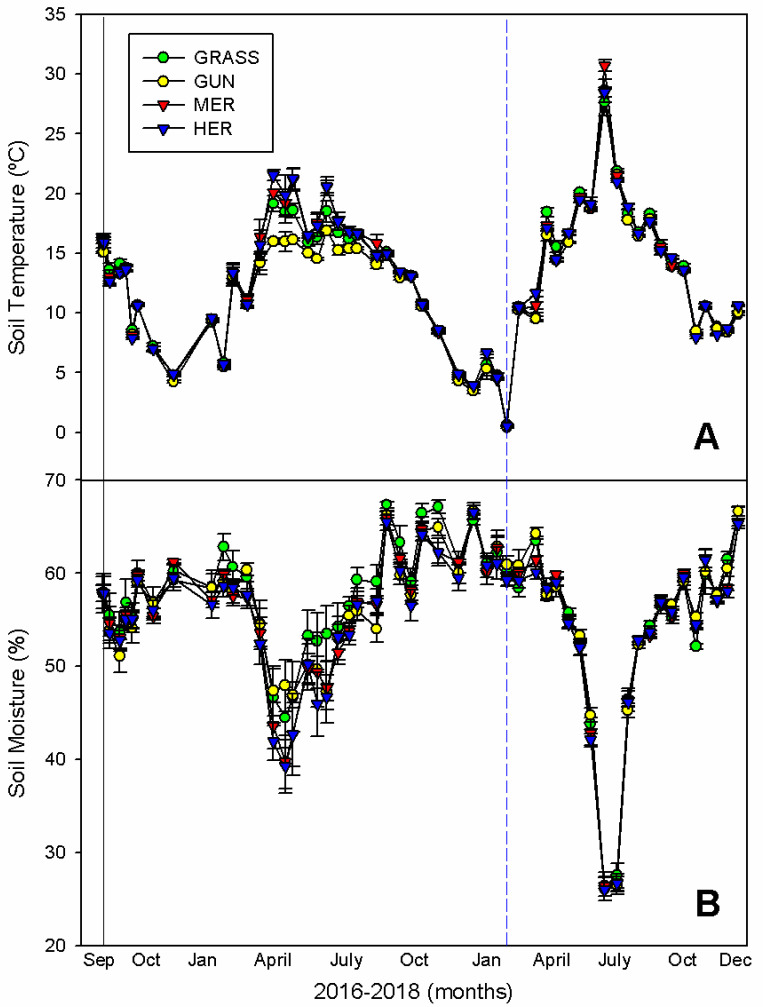
Seasonal variation in (**A**) soil temperature (°C) and (**B**) soil moisture (%) in invaded and uninvaded areas and after the physical removal or herbicide treatment of *G. tinctoria* on Achill Island, Co., Mayo, Ireland (n = 5; mean ± SE). Legend: GRASS, uninvaded semi-natural grasslands; GUN, areas invaded by *G. tinctoria*; MER, mechanical removal; and HER, herbicide (glyphosate) application. Note: the black solid line represents the initial removal, performed in invaded areas on 29th of September 2016, and the blue dashed line represents the extreme weather event called Storm Emma, at the end of February and beginning of March 2018.

Soil temperature in the different treatments varied seasonally (F_(159,848)_ = 2.77; *p* < 0.001; Figure 4A), and with exception of the summer of 2017 when soils under *G. tinctoria* were on average 4 °C cooler than uninvaded grasslands (95% CI = −4.63, −1.70), mechanical removal (95% CI = −5.59, −2.61) and herbicide treatment (95% CI = −7.03, −4.06), there were no other differences between the four treatments. In comparison to 2017, the following year was associated with more extreme soil temperatures, varying from 0 °C due to the extreme weather event in February–March to above 30 °C in summer due to an unusual period of high temperature in July (Figure 4A). Soil moisture fluctuated markedly (F_(53,848)_ = 180.31; *p* < 0.001; Figure 4B) and although high soil moisture values were found during winter (65–70%), for the major part of the year the values remained relatively constant (50–60%), apart from some low values in summer 2018 due to the higher than normal temperatures. No differences were found between the four treatments (F_(159,848)_ = 1.01; *p* = 0.445; Figure 4B). 

**Figure 5:**
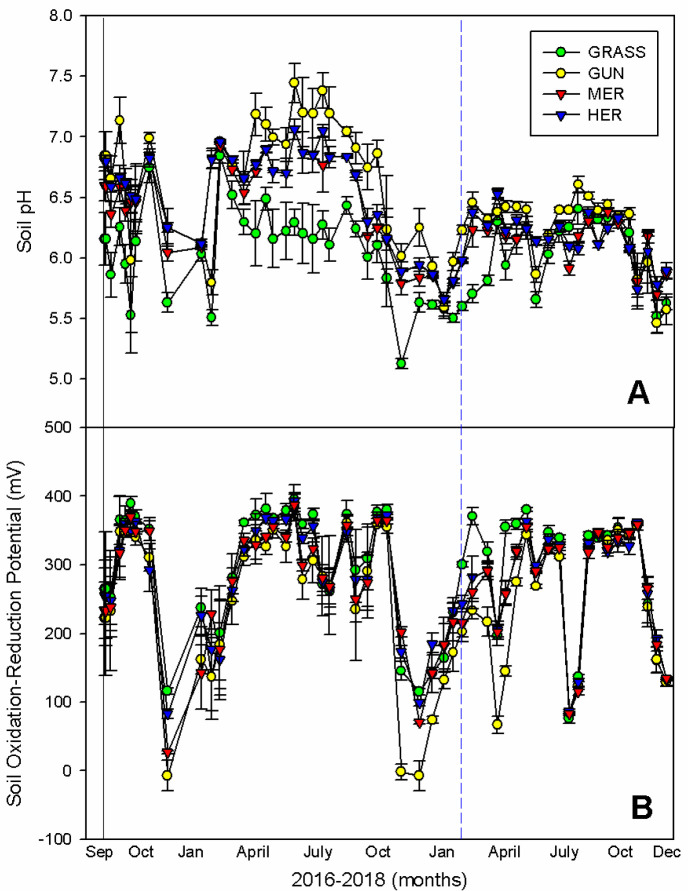
Seasonal variation in (**A**) soil pH and (**B**) soil oxidation-reduction potential in invaded and uninvaded areas and after the physical removal or herbicide treatment of *G. tinctoria* on Achill Island, Co., Mayo, Ireland (n = 5; mean ± SE). Legend: GRASS, uninvaded semi-natural grasslands; GUN, areas invaded by *G. tinctoria*; MER, mechanical removal; and HER, herbicide (glyphosate) application. Note: the black solid line represents the initial removal, performed in invaded areas on 29th of September 2016, and the blue dashed line represents the extreme weather event called Storm Emma, at the end of February and beginning of March 2018.

Soil pH (F_(159,848)_ = 2.65; *p* < 0.001; Figure 5A) and soil oxidation-reduction potential (F_(159,848)_ = 1.18; *p* < 0.001; Figure 5B) were influenced by *G. tinctoria* invasions with, on average, a one pH unit higher than uninvaded areas for the major part of the growing season in 2017 and lower-to-negative redox values, particularly in winter, in invaded areas. The legacy of the pH effect is evident in the removal and herbicide treatments which initially had a higher pH than the uninvaded area. After the extreme weather event all treatments had similar pH values (Figure 5A). 

## 3. Discussion

The smaller number of regenerating species in areas invaded by *G. tinctoria* was expected as this has been reported previously [29] and is a common feature associated with many plant invasions [35]. However, since *G. tinctoria* invasions are known to significantly deplete the soil seed bank [29], we did not expect such a rapid increase in species abundance and richness to values comparable with uninvaded areas after two years post-invasion. This was not consistent with our original hypothesis that there would be poor recovery of the plant community after physical removal or herbicide treatment of *G. tinctoria*. Whilst some recruitment could have been due to seed dispersal from neighbouring areas, most new individuals would have originated from the soil seed bank. This indicates that many Irish coastal grasslands have the potential to recover quickly after the removal of *G. tinctoria*, encouraging restoration measures in these important habitats. However, the generality of this effect is unclear as the magnitude of the impact of this plant invader on the seed bank is site-dependent [27,29] and a sufficient reservoir of viable seeds may not be present in all locations. Other factors, such as those related to seed size and nutrient reserves, seed dormancy and germination drivers, as well as life-forms, may also alter the outcomes of management decisions in areas colonised by other plant species, so that they may not necessarily show the same responses and post-invasion recovery characteristics as observed in this work.

The mechanical removal of mature *G. tinctoria* plants also resulted in a significant recruitment of a high number of young seedlings which is problematic and might require the implementation of additional management interventions. This was presumably a result of the increased exposure of buried seeds due to soil disturbance during the physical removal of mature plants. To this extent this supports our hypothesis that herbicide applications would be more effective in terms of post-invasion recovery of the community. There is also evidence that the germination of *G. tinctoria* seeds is less sensitive to light than the dominant co-occurring grass species *A. stolonifera* [36] and would have promoted their development. Interestingly, there was also evidence that the extreme weather event promoted seed germination of both the native species and *G. tinctoria*, particularly in plots where plants were physically removed and where more seeds were likely to be exposed to low temperatures. This was evident for *J. effusus* where there was an increase in both its abundance and cover after Storm Emma. However, the absence of any differences between the herbicide and mechanical removal plots indicates that for *J. effusus* this was not related to soil disturbance and may be a result of reduced competition from *G. tinctoria* plants, demonstrating the differential effect of disturbance on native and invasive plant species, as discussed for other extreme weather events [8,9,10]. This, therefore, supports our hypothesis that extreme weather events should favour native species such as *J. effusus*. However, the generality of this is uncertain, and based on the evidence provided in this study, the impact may depend on the developmental stage of the introduced species, as seedlings of *G. tinctoria* were less affected by low temperatures than mature plants. 

Whilst some of the effects of the extreme weather event were predictable, including a major reduction in the ability of mature established plants to regenerate new shoots, most of these plants survived (i.e., 29 ± 2% retained some shoots and 27 ± 3% resprouted after losing all their above-ground shoots). The other contribution (i.e., 44 ± 23%) to the total abundance of *G. tinctoria* was due to the presence of new individuals that germinated after Storm Emma had passed. It was also evident that the extreme weather event had a greater impact on shoot production by mature established plants than on seedlings. For instance, the total leaf area of mature plants was 11-fold smaller in comparison to the values in invaded plots prior to Storm Emma, whilst for seedlings, there was a large (i.e., 18-fold) increase [37]. This supports evidence that *G. tinctoria* is more tolerant of low temperatures than originally envisaged [27,33]. Nonetheless, other extreme weather events, such as severe episodic droughts, which are known to have significant impacts on many plant populations [8,9,10,19], might have a greater and more long-lasting impact on *G. tinctoria* given its susceptibility to water deficits and its high demand for water [27]. Although most mature plants lost their above-ground shoots and appear to be more susceptible to low temperatures than native species in the shorter-term, consistent with our original hypothesis, this needs to be tempered by the fact that most plants recovered. The results for seedlings contrast with our original hypothesis, as these were largely unaffected by the extreme weather event, indicating that the developmental stage is important. The fact that seedlings were less affected by low temperatures compared to mature plants suggests that the increased susceptibility observed in large plants is related to an effect on the initiation of new shoots from the rhizome apex, as well as the expansion of newly developed leaves, given the dramatic reduction in leaf area.

However, the phenology of mature *G. tinctoria* plants was altered and there was a delay of around six months before shoot production approached that prior to the occurrence of Storm Emma [37]. This evidence reinforces the idea that by taking advantage of the environmental conditions that compromise the performance of invasive plants this can provide an important window for reinforcing any earlier treatments or an opportunity for implementing additional eradication measures [6]. In terms of the recovery of the plant community, the removal of *G. tinctoria* plants increased the community similarity across all the treatments (of *c.* 60%). However, the greatest similarity (above *c.* 70%) was only found after the impact of the extreme weather event. Clearly, in the absence of the same or similar weather events the number of native species found in areas where *G. tinctoria* was removed or treated with herbicide would likely remain lower than in uninvaded areas even after two years. 

The idea of extreme weather events as drivers of changes in plant communities has been suggested previously [8,9,10,11,12,13]. The current study, however, provides empirical evidence that the impact of an extreme event can have significant effects on plant community dynamics by acting as an additional constraint to plant growth. Management interventions should, therefore, be cognizant of both the type of management that is implemented alongside their potential interactions with environmental factors. Although the impact of this extreme weather event on established plants of *G. tinctoria* did lead to significant reductions in shoot production and the death of some plants, this was transient, and an almost complete recovery of plant cover was found after a further two years [38].

The removal of *G. tinctoria* plants or their treatment with herbicide and the subsequent recovery of the original vegetation, allows us to examine the mechanisms by which this invasive species excludes many native species. Overall, the microclimatic effects of *G. tinctoria* on soil temperature and moisture were small apart from a significant reduction in soil temperature and an increase in soil moisture in 2018 during a period of unusually elevated temperatures, and these are, therefore, unlikely to be consistent drivers of any change in the plant community. In contrast, the presence of *G. tinctoria* was responsible for a consistent reduction in the soil oxidation-reduction potential and an increase in the soil pH, both of which recovered to pre-invasion conditions after removal of the plant invader or by the extreme weather event. Consequently, soil pH and redox potential might be factors contributing to the exclusion of some plant species from the standing vegetation. Whilst it is known that soil legacy effects can impact on the regeneration of native species and act as a barrier to restoration [39], most of the effects on soil pH/oxidation-reduction potential were transient and disappeared after the removal of *G. tinctoria* plants. The impacts of an extreme weather event on soil legacy effects may, however, depend on their relative impact on the invasive species, as well as the type of soil legacy effect being studied, and this needs further investigation.

Although other factors that were not measured in this study, such as soil water table variations or alterations in soil cationic exchange capacity, could have contributed to the exclusion of native species from stands of *G. tinctoria*, the most likely mechanism is due to a shading effect. Only the most shade tolerant species are likely to be able to germinate and complete their development underneath mature stands of *G. tinctoria*, where most incident light is excluded from early in the growing season, prior to the initiation of growth of most native species, and lasting for approximately eight months of the year [40]. This proposal is supported by the evidence that native species that are common in exposed situations (i.e., open areas with high light levels), such as *C. palustre*, *J. articulatus*, *L. salicaria*, *M. aquatica, Potentilla* spp., *P. vulgaris*, *Rumex* spp. and *T. repens*, and a number of common grass species found in Irish grasslands, such as, *F. ovina* and *H. lanatus*, were only present in previously invaded plots after the additional impact of the extreme weather event, which resulted in further reductions in the performance of *G. tinctoria* (Table 2). This led to an increase in the germination of many light demanding seeds that are still present in the seed bank, as well as stimulating the growth of *J. effusus*. The extreme weather event, therefore, acted like a removal protocol since this initially killed most emerging leaves/shoots of the invader and reduced light interception by 45%, resulting in increased seed germination [37].

In conclusion, mechanical removal, or herbicide application of mature *G. tinctoria* plants can result in the near complete restoration of the original community within two years, with additional benefits associated with an extreme event. In the longer-term, however, this may be compromised by disturbance-related increases in the emergence of seedlings of the invader, as seen in the mechanical removal plots. Additionally, some differences in plant community composition remained two years after the implementation of these management interventions. For instance, *A. odoratum*, *C. stagnalis* and *F. ulmaria* were only present in uninvaded areas, even after Storm Emma, indicating that some native species might need a longer time to recolonise areas where *G. tinctoria* was removed. 

Surprisingly, the near complete restoration of the plant community occurred despite evidence of a major reduction in seed numbers and biodiversity in the seed bank [27,29]. This indicates that there is still a sufficient pool of viable seeds in the soil seed bank to restore much of the original plant community, even after more than 50 years since the establishment of *G. tinctoria* introductions [41]. Although both removal protocols have a similar impact in terms of the regeneration of native species, mechanical removal of mature plants significantly increased the numbers of regenerating *G. tinctoria* seedlings, which may necessitate further management interventions. Whilst extreme climatic events are generally considered a particularly detrimental aspect of climate change, the results of this study indicate that they may contribute beneficially to the restoration of communities invaded by *G. tinctoria*, providing a window of opportunity to implement repeat or new management measures.

## 4. Material and Methods

### 4.1. Area of Study and Experimental Design

Five fields were selected, comprising invasive populations and nearby uninvaded semi-natural grasslands, on Dooega Beach, Achill Island (53°51” and 54°01′ N; and 9°55′ and 10°15′ W), Co. Mayo, west coast of Ireland. The annual rainfall is above 1,100 mm and the temperature ranges from 3 °C to 6 °C in winter and from 12 °C to 15 °C in summer [27]. On Achill Island, *G. tinctoria* invades alluvial or colluvial soils, derived from volcanic material or thin gley soils of marine origin with a pH that ranges from 4.6 to 6.2 [27]. This field experiment study area has been reported previously [40,42].

In each field, we established four 5 × 5 m plots (totalling 20 plots, n = 5 for each treatment) which were allocated to the following treatments: (1) uninvaded semi-natural grasslands dominated by *J. effusus* (GRASS); (2) areas invaded by *G. tinctoria* and considered as controls (GUN); (3) areas within invasive populations of *G. tinctoria* where individuals were mechanically removed (MER); and (4) areas within invasive populations where plants were sprayed with glyphosate (HER). The distance between plots was *c.* 10 m and each replicate was at least 100 m apart from each other. The total area studied was 500 m^2^ or 0.05 ha.

### 4.2. Mechanical Removal and Herbicide Application

The mechanical removal and herbicide application were carried out at the end of September 2016 with a follow up in early April 2017 to remove resprouted material. These periods of the year are considered the most appropriate for controlling this plant invader [31]. Both mechanical removal and herbicide applications were implemented on an area that extended 1 m outside the plot boundary (5 × 5 m) to limit recolonisation from invaded areas. Strict protocols were followed to avoid dispersing any plant material, including boot washing to remove any seeds or other propagules. 

For the mechanical removal, a machete was used to cut and remove the leaves and petioles to get access to the rhizomes. After this, a shovel was used to first slice and then to dig out the rhizomes and roots to a depth of *c.* 30 cm, with further sorting of soil to remove any fragments of plant material that were remaining. Whilst care was taken during the removal process, some soil disturbance was unavoidable. For the herbicide applications, leaves of *G. tinctoria* were sprayed with RoundUp Flex (Monsanto ^®^) using a knapsack sprayer following the manufacturer’s recommendations (concentration of 4 L.ha^−1^) [43]. Herbicide spraying was carried out in the morning on sunny days when there were light-to-no winds to avoid herbicide drifting to adjacent areas. 

### 4.3. Species Richness and Plant Community Similarity

To assess species richness (i.e., presence or absence only) we carried out an inventory every three months between 2016–2018 at 0 (before removal), 3, 6, 9, 12, 15, 18, 21 and 24 months after the initial removal to examine seasonal changes in the vegetation (i.e., January, April, July and October, referred to as winter, spring, summer and autumn). To compare differences between the communities amongst the four treatments caused by the removal protocols and the extreme weather event, the Jaccard’s similarity index was used. We decided to use Jaccard’s index since it is one of the most commonly used similarity tests for assessing beta diversity with presence and absence data. Although there are newer approaches, this method is still valid given that we were more interested in the final similarity of the communities between treatments rather than in the species turnover itself [44]. Community similarity indexes are described in Table 1. The species identified prior to, one year after and two years after the mechanical removal and herbicide treatments were implemented are shown in Table 2. 

### 4.4. Plant Abundance and Cover

We determined the abundance of *G. tinctoria* and the abundance and percentage cover of *J. effusus*. We focused on *J. effusus* for comparison with *G. tinctoria* as invasions resulted in a reduction of more than 97% of its abundance (ANOVA, F_(1,8)_ = 18.182; *p* = 0.003) in previous assessments (data not shown) [37]. To estimate the abundance of *J. effusus*, clearly separated individual tussocks were considered as one single individual; when the separation of individuals could not be done with confidence, we also considered them as one single individual. To estimate the cover of *J. effusus*, we made visual assessments using discrete units of 5%. For this, we divided the 5 × 5 m plots into four smaller quadrats using a measuring tape and attributed them a cover percentage as estimated by eye. The percentage attributed to each of the four smaller quadrats was then summed up and averaged to obtain a value for the whole plot. 

To estimate the abundance of mature *G. tinctoria* plants, we counted the number of plants inside all plots, considering as one single individual when the rhizomes were clearly separated. When rhizomes had two apexes, but were connected, we considered it as a single individual. Seedlings were counted individually since they are easier to identify as they do not produce a significant rhizome in the first few years of growth [40]. To estimate the abundance of viable *G. tinctoria* plants after herbicide applications or the extreme weather event, we considered plants that had the capability to produce new shoots as alive. For *G. tinctoria* all above-ground structures arise from a region close to the rhizome apex and, when healthy, are surrounded by leaf-like reddish bracts [27,40]. Consequently, if the apex was brown and/or rotten and plants failed to produce any new shoots, the plant was considered to be dead. If plants produced new above-ground structures in other regions of the rhizome (e.g., the end of the rhizome), this was considered as a new individual. Consequently, if individuals did not produce any shoots and/or resprout, they were considered as dead. For total abundance, both mature established plants and seedlings of the plant invader were counted together. 

### 4.5. Environmental Data—Soil Variables and Storm Emma

Data for different environmental variables were collected every two weeks. Soil moisture and temperature data were collected using a WET-2 WET sensor (Delta-T Devices Ltd., Cambridge, UK), with the instrument’s trident probe inserted to a depth of *c.* 5–10 cm. To measure soil pH and soil oxidation-reduction potential we used a HI 9125 pH/ORP meter (Hanna Instruments ^®^, Woonsocket, RI, USA) inserted to a depth of *c.* 5 cm. All soil variables were estimated at three random positions within individual plots and were subsequently summed and averaged.

The extreme weather event, Storm Emma, occurred in late February to early March 2018 and affected Ireland as a whole. Temperatures dropped below 0 °C with snowfall over a major part of Ireland, during the early part of the growing season after the initiation of plant growth. Based on the 60-y historical average, the mean air temperatures for this period would be *c.* 7.5 °C [40], several degrees higher than those recorded during the extreme weather event. Whilst the eastern part of the country reported snow accumulation of up to 1.5 m, in the study area this was much smaller, with an accumulation of *c.* 30 cm, according to local information. As Storm Emma occurred after *G. tinctoria* plants had initiated leaf-out, the extreme weather event acted like a removal protocol, killing most leaves in invasive stands.

### 4.6. Statistical Analysis

We used general linear mixed-model effects analyses with restricted maximum likelihood to verify differences between treatments and their interaction with sampling times, considering the different treatments and fields as fixed and random factors, respectively. We evaluated differences in sampling times (0, or before removal, and then at 3, 6, 9, 12, 15, 18, 21 and 24 months afterwards) on species richness, abundance and cover. The Bonferroni’s post hoc correction was used to perform pair-wise comparisons and visual analysis of the residuals was also carried out to ensure normality. Abundance data for *G. tinctoria* and *J. effusus* were Log (x + 1) transformed, and *J. effusus* cover and associated soil moisture data were arcsine square root transformed prior to analysis. All analyses were done considering *p* = 0.05, using SPSS Statistics v. 24 [45].

## Figures and Tables

**Table 1:** Similarity of plant communities (%) according to the Jaccard Index for treatments (n = 5), on Achill Island, Co., Mayo, Ireland. Legend: Time 0 M indicates the time prior to the implementation of the mechanical removal or herbicide application on 29th September 2016 and Time 24 M indicates two years after the treatments had been applied; GRASS, uninvaded semi-natural grasslands; GUN, areas invaded by *G. tinctoria*; MER, mechanical removal; and HER, herbicide (glyphosate) application.

Similarity (%) per Treatment		Time 0 M				Time 24 M			
		GRASS	GUN	MER	HER	GRASS	GUN	MER	HER
**Time 0 M**	**GRASS**	-	-	-	-	-	-	-	-
	**GUN**	18.18	-	-	-	-	-	-	-
	**MER**	15.91	54.55	-	-	-	-	-	-
	**HER**	13.64	60.00	66.67	-	-	-	-	-
**Time 24 M**	**GRASS**	79.63	-	-	-	-	-	-	-
	**GUN**	-	21.43	-	-	77.78	-	-	-
	**MER**	-	-	16.67	-	82.14	87.50	-	-
	**HER**	-	-	-	14.29	83.93	82.00	94.00	-

**Table 2:** List of species identified in all treatments throughout 2016–2018, on Achill Island, Co., Mayo, Ireland. Note: Time 0 M indicates the time prior to the implementation of the mechanical removal or herbicide application on 29th September 2016; Time 12 M is after one year and Time 24 M is two years after the treatments had been applied. The extreme weather event called Storm Emma occurred at the end of February/beginning of March 2018, prior to the assessments made at Time 24 M. Legend: GRASS, uninvaded semi-natural grasslands; GUN, areas invaded by *Gunnera tinctoria*; MER, mechanical removal; and HER, herbicide (glyphosate) application.

Scientific Name	Botanical Family	Time 0 M				Time 12 M				Time 24 M			
		GRASS	GUN	MER	HER	GRASS	GUN	MER	HER	GRASS	GUN	MER	HER
*Angelica sylvestris* L.	Apiaceae	X				X	X	X	X	X	X	X	X
*Apium nodiflorum* (L.) Lag.		X				X	X	X	X	X	X	X	X
*Berula erecta* (Huds.) Coville		X				X				X			
*Daucus carota* L.												X	X
*Cirsium palustre* (L.) Scop.	Asteraceae	X				X		X	X	X	X	X	X
*Cirsium vulgare* (Savi) Ten.		X				X	X	X	X	X	X	X	X
*Senecio jacobaea* L.										X	X	X	X
*Taraxacum officinale* F.H. Wigg.		X				X		X	X	X	X	X	X
*Myosotis secunda* A. Murray	Boraginaceae									X	X	X	X
*Cardamine hirsuta* L.	Brassicaceae							X					
*Cardamine pratensis* L.										X			
*Cerastium fontanum* Baumg.	Caryophyllaceae	X							X	X		X	X
*Sagina procumbens* L.		X				X				X			
*Carex echinata* Murray	Cyperaceae												X
*Dryopteris filix-mas* (L.) Schoott	Dryopteridaceae	X				X			X	X			X
*Equisetum arvense* L.	Equisetaceae	X	X			X	X	X	X	X	X	X	X
*Equisetum fluviatile* L.		X	X			X	X		X	X	X	X	X
*Trifolium pratense* L.	Fabaceae	X				X				X		X	X
*Trifolium repens* L.		X				X		X	X	X	X	X	X
*Gunnera tinctoria* Molina (Mirb.)	Gunneraceae		X	X	X	X	X	X	X	X	X	X	X
*Crocosmia x crocosmiiflora* (Lemoine) N.E. Br.	Iridaceae	X				X	X	X	X	X	X	X	X
*Juncus acutiflorus* Ehrh.	Juncaceae	X				X				X		X	X
*Juncus articulatus* L.		X				X		X	X	X	X	X	X
*Juncus effusus* L.		X	X	X		X	X	X	X	X	X	X	X
*Mentha aquatica* L.	Lamiaceae	X				X		X	X	X	X	X	X
*Prunella vulgaris* L.		X				X				X	X	X	X
*Lythrum salicaria* L.	Lythraceae	X				X		X	X	X	X	X	X
*Epilobium obscurum* Schreb	Onagraceae	X	X	X	X	X	X	X	X	X	X	X	X
*Callitriche stagnalis* Scop.	Plantaginaceae	X				X				X			
*Plantago lanceolata* L.		X				X	X	X	X	X	X	X	X
*Veronica beccabunga* L.		X				X		X		X	X	X	X
*Agrostis capillaris* L.	Poaceae	X	X	X	X	X	X	X	X	X	X	X	X
*Agrostis stolonifera* L.		X	X	X	X	X	X	X	X	X	X	X	X
*Alopecurus pratensis* L.		X				X		X	X	X		X	X
*Anthoxanthum odoratum* L.										X			
*Elymus repens* (L.) Gould								X					
*Festuca ovina* L.		X				X		X	X	X	X	X	X
*Holcus lanatus* L.		X				X				X	X	X	X
*Holcus mollis* L.								X					
*Lolium perenne* L.		X				X				X		X	X
*Paneion pratense* (L.) Lunell		X				X		X	X	X	X	X	X
*Poa annua* L.		X				X	X	X	X	X	X	X	X
*Poa trivialis* L.		X				X	X	X	X	X	X	X	X
*Persicaria maculosa* Gray	Polygonaceae									X	X	X	X
*Rumex acetosa* L.		X				X				X	X	X	X
*Rumex conglomeratus* L.		X				X	X	X	X	X	X	X	X
*Rumex crispus* L.								X		X	X	X	X
*Rumex obtusifolius* L.		X				X	X	X	X	X	X	X	X
*Anagallis tenella* (L.) L.	Primulaceae									X	X	X	
*Ranunculus acris* L.	Ranunculaceae	X		X		X	X	X	X	X	X	X	X
*Ranunculus repens* L.		X	X		X	X	X	X	X	X	X	X	X
*Filipendula ulmaria* (L.) Maxim.	Rosaceae									X			
*Potentilla anserina* L.		X				X		X	X	X	X	X	X
*Potentilla erecta* (L.) Raeusch.										X	X	X	X
*Potentilla reptans* L.								X		X	X	X	X
*Rubus fruticosus* L.		X				X	X	X	X	X	X	X	X
*Galium aparine* L.	Rubiaceae	X		X	X	X	X	X	X	X	X	X	X
*Galium palustre* L.		X				X		X	X	X	X	X	X
*Urtica dioica* L.	Urticaceae	X	X	X	X	X	X	X	X	X	X	X	X

## Data Availability

The data presented in this study are available on request from the corresponding author. The data are not publicly available due to limitations of consent requested by the participants of the study and landowners where the fieldwork experiment was done.
